# The Potential Protective Effects of Polyphenols in Asbestos-Mediated Inflammation and Carcinogenesis of Mesothelium

**DOI:** 10.3390/nu8050275

**Published:** 2016-05-09

**Authors:** Monica Benvenuto, Rosanna Mattera, Gloria Taffera, Maria Gabriella Giganti, Paolo Lido, Laura Masuelli, Andrea Modesti, Roberto Bei

**Affiliations:** 1 Department of Clinical Sciences and Translational Medicine, University of Rome “Tor Vergata”, Rome 00133, Italy; monicab4@hotmail.it (M.B.); rosannamatter@gmail.com (R.M.); g.taffera@gmail.com (G.T.); giganti@med.uniroma2.it (M.G.G.); modesti@med.uniroma2.it (A.M.); 2 Internal Medicine Residency Program, University of Rome “Tor Vergata”, Rome 00133, Italy; paulshore@virgilio.it; 3 Department of Experimental Medicine, University of Rome “Sapienza”, Rome 00164, Italy; Laura.masuelli@uniroma1.it

**Keywords:** malignant mesothelioma, inflammation, immune system, ROS and RNS, polyphenols, asbestos

## Abstract

Malignant Mesothelioma (MM) is a tumor of the serous membranes linked to exposure to asbestos. A chronic inflammatory response orchestrated by mesothelial cells contributes to the development and progression of MM. The evidence that: (a) multiple signaling pathways are aberrantly activated in MM cells; (b) asbestos mediated-chronic inflammation has a key role in MM carcinogenesis; (c) the deregulation of the immune system might favor the development of MM; and (d) a drug might have a better efficacy when injected into a serous cavity thus bypassing biotransformation and reaching an effective dose has prompted investigations to evaluate the effects of polyphenols for the therapy and prevention of MM. Dietary polyphenols are able to inhibit cancer cell growth by targeting multiple signaling pathways, reducing inflammation, and modulating immune response. The ability of polyphenols to modulate the production of pro-inflammatory molecules by targeting signaling pathways or ROS might represent a key mechanism to prevent and/or to contrast the development of MM. In this review, we will report the current knowledge on the ability of polyphenols to modulate the immune system and production of mediators of inflammation, thus revealing an important tool in preventing and/or counteracting the growth of MM.

## 1. Introduction

Malignant Mesothelioma (MM) is a rare primary tumor arising from the mesothelial cell linings of the serous membranes, most commonly involving the pleural and peritoneal spaces [1]. The development of MM consists of a multi-step process driven by cellular DNA damage and tumor cell promotion, in which genetically modified mesothelial cells are prone to grow, and tumor progression, in which mesothelial cells develop a more aggressive phenotype and eventually acquire the ability to metastasize and invade other tissues. The immune system’s involvement in the development of MM is complex and multifaceted and is likely to involve both the innate and adaptive immune systems [2,3,4]. A chronic inflammatory response orchestrated by mesothelial cells contributes to the development and progression of mesothelial cells into MM [2,3,4]. 

Cisplatin and antifolate-based combination chemotherapy represent the standard first-line treatment for advanced and unresectable MM patients [5]. However, taking into account the poor outcome and toxicity of chemotherapy, novel approaches based on targeting abnormally activated signaling pathways in MM cells were employed to improve survival in MM patients, as described in the review by Remon *et al.* [5]. Clinical trials have employed antiangiogenic and vascular disrupting agents, PI3K/AKT/mTOR pathway inhibitors, heat shock protein S90 inhibitors and arginine depletory molecule, and immunotherapy. However, although some of these clinical trials sustain further studies, the absolute response rates (RRs) are limited compared to other tumors [5].

The knowledge of MM pathophysiology might influence novel approaches [6,7,8,9]. 

The evidence that: (a) multiple signaling pathways are aberrantly activated in MM cells [10]; (b) asbestos-mediated chronic inflammation through the release of reactive oxygen species (ROS), nitrogen species (RNS) and cytokines has a key role in MM carcinogenesis [11]; (c) the deregulation of the immune system might favor the onset of MM [9]; and (d) a drug might have a better efficacy when injected into a serous cavity, thus bypassing biotransformation and reaching an effective dose [12], have prompted investigations to evaluate the effects of polyphenols for the therapy and prevention of MM. Dietary polyphenols possess pleiotropic properties capable of being able to (a) inhibit cancer cell growth by targeting multiple signaling pathways; (b) reduce inflammation and (c) modulate immune response [13,14,15,16,17].

The ability to reduce chronic inflammation might represent a key mechanism to contrast the development and/or to prevent MM. Accordingly, the local or systemic administration of polyphenols might reduce the production of pro-inflammatory molecules by targeting signal transduction pathways or ROS and RNS. In addition, the pro-oxidant activity of polyphenols could be a strategy to kill cancer cells and thus to limit tumor growth [14]. 

In this review we will report the current knowledge on the ability of polyphenols to modulate the immune system and production of mediators of inflammation in MM, thus revealing an important tool to prevent and/or to counteract the growth of MM. 

## 2. Polyphenols

Polyphenols, a large group of phytochemicals ubiquitously found in plants, are secondary metabolites that perform functions in the host’s defense against pathogens, ultraviolet radiation, and signal transduction [18]. Polyphenols are present in food and beverages of plant origin, such as fruits, vegetables, cereals, spices, legumes, nuts, olives, tea, coffee, and wine [19]. These compounds exhibit anti-inflammatory, antimicrobial, anticancer, and immunomodulatory activities, and thus are beneficial for human health [20]. 

Polyphenols have a characteristic phenolic structure and are classified according to the number of phenol rings that they contain and by the structural elements that bind these rings to one another. The main classes of polyphenols are flavonoids, phenolic acids, stilbenes, and lignans [18,21]. 

Among flavonoids, the most important subclasses are flavonols, flavones, flavan-3-ols, anthocyanins, flavanones, and isoflavones. The flavonoid subclasses dihydroflavonols, flavan-3,4-diols, chalcones, dihydrochalcones, and aurones are minor components of our diet [22]. 

Quercetin, kaempferol, and myricetin, found mostly in fruits, edible plants, wine, and tea, are the main flavonols [18]. The most abundant flavones in foods are apigenin (parsley, celery, onion, garlic, pepper, chamomile tea) and luteolin (Thai chili, onion leaves, celery) [20]. The flavan-3-ol subclass includes a wide range of compounds with different chemical structures that can be divided in monomers, (+)-catechin, (−)-epicatechin, (+)-gallocatechin, (−)-epigallocatechin, (−)-epicatechin-3-*O*-gallate, (−)-epigallocatechin-3-*O-*gallate, and polymers (proanthocyanidins) and are found mainly in fruits, berries, cereals, nuts, chocolate, red wine, and tea [20]. The most abundant anthocyanins (cyanidin, pelargonidin, delphinidin, peonidin, petunidin, and malvidin) are found mainly in berries, cherries, red grapes, and currants [23]. Flavanones are present in citrus fruit and the most important are hesperetin and naringenin and their correspondent glycated forms (naringin (naringenin-7-*O*-neohesperidoside), neohesperidin (hesperetin-7-*O*-neohesperidoside), narirutin (naringenin-7-*O*-rutinoside), and hesperidin (hesperetin-7-*O*-rutinoside)) [21,24]. Daidzein, genistein, and glyciten are the most common members of isoflavones, found mainly in soybeans, soy products, and leguminous plants [25]. 

Among phenolic acids, the hydroxybenzoic acids (protocatechuic acid and gallic acid) are found in few edible plants, while hydroxycinnamic acids (caffeic acid, ferulic acid, *p*-coumaric acid, and sinapic acid) are found in fruits, coffee, and cereal grains [18].

The main member of stilbenes is resveratrol (3,5,4′-trihydroxystilbene) and is present in grapes, berries, plums, peanuts, and pine nuts [21]. Lignans (ecoisolariciresinol, matairesinol, medioresinol, pinoresinol, and lariciresinol) are found in high concentration in linseed and in minor concentration in algae, leguminous plants, cereals, vegetables, and fruits [18]. Curcumin (1,7-bis-(4-hydroxy-3-methoxyphenyl)-1,6-heptadiene-3,5-dione), a member of the curcuminoid family, is another polyphenol compound found in turmeric, a spice produced from the rhizome of *Curcuma* *longa* [26].

Polyphenols have important anti-inflammatory effects by regulating innate and adaptive immunity through the modulation of different cytokines and also by acting as an immune surveillance mechanism against cancers through the regulation of apoptosis [14]. Polyphenols also possess anti-oxidant and pro-oxydant activities [27,28,29,30] and are able to modulate multiple targets involved in carcinogenesis through simultaneous direct interaction or modulation of gene expression [13]. It is worth nothing that these compounds are able to inhibit the growth of cancer cells without having an adverse effect on normal cells. In this way, polyphenols play selectively an antitumor role in cancer [14]. However, despite promising results obtained from *in vitro* studies, the use of polyphenols as anticancer agents is yet limited in clinical practice due to their low bioavailability in the human body, which affects the effective dose delivered to cancer cells. In fact, polyphenols have a poor absorption and biodistribution and a fast metabolism and excretion in the human body. Only nano- or micromolar concentrations of polyphenols and polyphenol metabolites are found in plasma (0–4 μM after an intake of 50 mg of aglycone equivalents) [31]. Several mechanisms limit the bioavailability of polyphenols, including their metabolism in the gastrointestinal tract and liver, their binding on the surfaces of blood cells and microbial flora in the oral cavity and gut, and regulatory mechanisms that prevent the toxic effects of high compound levels on mitochondria or other organelles [32]. In addition to endogenous factors, dietary factors can affect the bioavailability of polyphenols, such as food matrix and food preparation techniques [33]. Promising strategies for improving the *in vivo* anticancer effects of polyphenols are the combination of polyphenols, or polyphenols and conventional cancer treatments, and the intratumoral administration of polyphenols, in order to bypass biotransformation and reach an effective dose directly available at the site of tumor [22]. Several pre-clinical and Phase I and Phase II clinical trials are employing intratumoral administration to deliver different therapeutics such as drugs, viral-based cancer vaccines, immune cell-based vaccines, cytokines, DNA, bacterial products, nanoparticles, and natural compounds to the tumor site [34,35,36,37,38,39]. Thus, intratumoral delivery of cancer therapeutics could be a more efficient route of administration for several agents in easily accessible tumors, such as MM. An intratumoral route of administration is able to prevent the occurrence of systemic side effects and makes the therapeutic agents directly available at the tumor site, allowing for the highest concentration close to tumor cells [40]. 

## 3. Asbestos Fibers and MM

MM was broadly observed in the mid-to-late 1960s among workers whose asbestos exposure began 30–40 years earlier [41]. Accordingly, the development of MM has been linked to exposure to asbestos fibers [1]. Although the use of asbestos has by now been prohibited in 55 countries, the occurrence of asbestos-related diseases cannot decrease due to: the long latency period of MM, the continued use of asbestos in Third World Countries and the continued occupational exposure in Western Countries, such as the US and Europe [5,42].

Thermo-resistant magnesium and calcium silicate fibers were usually used as insulating materials in buildings and are deposited in the alveoli upon inhalation. Asbestos fibers are genotoxic, causing random chromosome breaks [43]. Asbestos is classified into two major categories (amphibole and serpentine) [43]. There are five members of the amphibole category: crocidolite (blue asbestos), amosite (brown asbestos), tremolite, anthophyllite, and actinolite. The serpentine class is made up of only one member (chrysotile, white asbestos) [43]. The International Agency for Research on Cancer (IARC) classifies asbestos as a Group I carcinogen, because of the ability of chrysotile, crocidolite, and amosite to induce lung cancer or MM [44]. Fiber translocation into the pleural cavity can occur across the alveolar surface or via pulmonary lymph flow [45]. 

Although the link between MM and asbestos is well established, other carcinogenic or co-carcinogenic events must be involved in MM development because only 10% of all MM cases occur in asbestos-exposed subjects [46]. 

## 4. Chronic Inflammation Affects MM Development 

### 4.1. Overproduction of ROS and RNS

A key immune-mediated involvement in asbestos-related carcinogenesis is superimposed on the fibers’ damage to the mesothelium integrity. Mesothelial cells offer the first defense against chemical and biological injuries by building a mechanical barrier and also by activating inflammation through the release of ROS, RNS, and cytokines [47,48,49,50,51]. In fact, mesothelial cells express on their luminal surface a sialomucins veil that electrostatically repels bacteria, viruses, and chemicals and mechanically decreases their adherence to the mesothelial layer [47]. In addition, serous spaces are surrounded by several defensive molecules including lysozyme, IgA immunoglobulins, and complement factors [47]. Mesothelial cells damaged by asbestos fibers release inflammatory mediators that maintain an inflammatory environment [48,49,50]. 

Asbestos induces free radical production by mesothelial cells through the iron content of the asbestos fibers which increases the hydroxyl radical formation from hydrogen peroxide through iron catalysed reactions and by inflammatory cells such as pulmonary alveolar macrophages and neutrophils [52]. Kinnula *et al.* reported that inflammatory cells are the essential cells responsible for the free radical-mediated mesothelial cell injury during asbestos exposure *in vivo* [53,54]. During the respiratory burst, leukocytes produce multiple ROS, including hydroxyl radical, superoxide anions, and hydrogen peroxide [55]. Hansen *et al.* reported that the geometry and/or chemical composition of asbestos is important for the release of superoxide anions by leucocytes during frustrated phagocytosis [56,57]. Indeed, crocidolite and amosite induced significant ROS generation by neutrophils with a peak at 10 min, whereas that of chrysotile was ~25% of the crocidolite/amosite response [58]. 

Leukocytes and mesothelial cells are also able to overexpress nitric oxide synthase (NOS) in response to a variety of stimuli [52]. Inflammatory cytokines and oxidant stress can each augment iNOS expression and activity in pulmonary alveolar epithelial cells [52]. The inhalation of either chrysotile or crocidolite asbestos fibers was shown to induce the production of nitric oxide in bronchoalveolar lavage cells and the formation of nitrotyrosine within the lungs and pleura [59]. The majority of MM was found to express high levels of iNOS, while its expression was occasionally found in non-neoplastic healthy mesothelium [60]. Thus, RNS might have an important role in asbestos-mediated mesothelioma oncogenesis [52]. Peroxynitrite can be produced by the reaction of ROS with RNS. The overproduction of ROS and RNS in the inflammatory microenvironment can cause DNA damage to mesothelial cells, thus leading to the development of MM [61].

Macrophages are recruited and activated to clear away asbestos fibers [48]. Vitronectin captures crocidolite asbestos and enhances fiber phagocytosis by mesothelial cells via integrins [62]. Yang *et al.* provided the mechanistic rationale that associates asbestos-mediated mesothelial cell necrosis to the chronic inflammatory reaction that is, in turn, linked with asbestos-mediated tumorigenesis [63]. The authors reported that exposure of mesothelial cells to crocidolite asbestos induces them to activate poly(ADP-ribose) polymerase, to secrete hydrogen peroxide, to deplete ATP, and to secrete high-mobility group box 1 protein (HMGB1) into the extracellular space. This latter stimulates macrophages to secrete tumor necrosis factor-α (TNF-α) and the inflammatory response associated with asbestos-mediated carcinogenesis [2]. 

The asbestos-mediated chronic inflammation can increase the genotoxic damage due to the secretion of free radicals [48]. 

**Figure 1:**
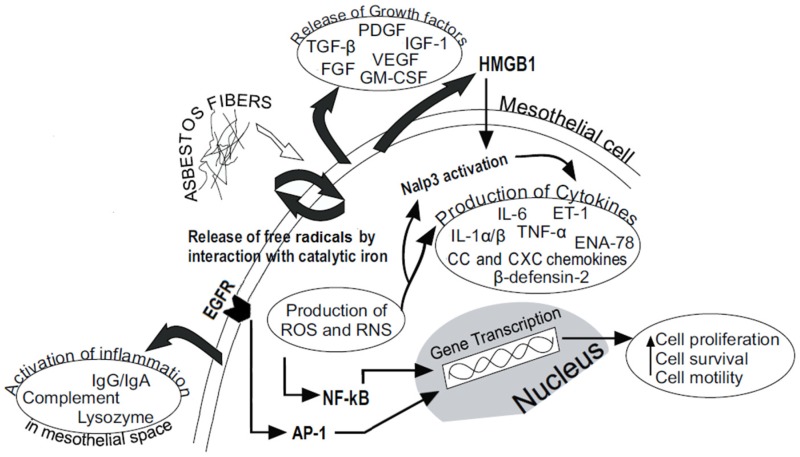
The asbestos-mediated long-lasting inflammation in mesothelial cells. Biological responses of mesothelial cells to asbestos fiber injury. Abbreviations: ROS, Reactive Oxygen Species; RNS, Reactive Nitrogen Species; HMGB1, High-Mobility Group Box 1 Protein; PDGF, Platelet-Derived Growth Factors; FGF, Fibroblast Growth Factor; IGF-1, Insulin-Like Growth Factor-1; VEGF, Vascular Endothelial Growth Factor; TGF-β, Transforming Growth Factor-β; GM-CSF, Granulocyte/Macrophage-Colony Stimulating Factor; IL-6, Interleukin-6; ET-1, Endothelin-1; IL-1 α/β, Interleukin-1 α/β; TNF-α, Tumor Necrosis Factor-α; ENA-78, Epithelial Neutrophil Activating Protein-78; NF-κB, Nuclear Factor-kB; EGFR, Epidermal Growth Factor Receptor; AP-1, Activator Protein-1.

In addition, several studies have shown that asbestos fibers induce the activation of EGFR (Epidermal growth factor receptor) and thus MAPK (Mitogen-activated protein kinase) pathway and AP-1 (Activator protein-1), leading to cell proliferation [43] (Figure 1). 

### 4.2. Inflammasome Activation and Cytokines Secretion 

It was reported that exposure to asbestos induces mesothelial cell necrosis and the release of HMGB1 into the extracellular space [2]. HMGB1 is a key mediator of chronic inflammation in MM, leading to Nalp3 inflammasome activation, macrophages accumulation, interleukin (IL)-1β and TNF-α secretion, and thus to activation of the NF-κB pathway, which increases cell survival and tumor growth after asbestos exposure [50]. A recent study reported that HMGB1 localization was regulated by its acetylation. In fact, HMGB1 is localized in the nucleus to stabilize nucleosomes when it is in the nonacetylated form. When HMGB1 is hyperacetylated, it is actively secreted into the extracellular space. The authors indicated that HMGB1 hyperacetylation could be a sensitive and specific biomarker to discriminate MM patients from asbestos-exposed individuals and from healthy unexposed controls. They demonstrated that hyperacetylated HMGB1 was significantly higher in MM patients compared with asbestos-exposed individuals and healthy controls, and did not vary with tumor stage [4].

Accordingly, asbestos fibers induce NLRP3 priming and activation, thus leading to increased transcription of pro-inflammatory cytokines [63,64]. The inflammasome is a constituent of the inflammation machinery which includes NOD-like receptors (NLRs) whose activation induces the activation of caspase-1 and of the mature form pro-inflammatory cytokines, such as IL-1β and IL-18 [65,66]. It was reported that the NLRP3 inflammasome is necessary for early inflammatory responses to asbestos, but it is not indispensable for asbestos-induced MM [64]. Hillegass *et al.* linked NLRP3 activation to the release of several pro-inflammatory cytokines (IL-1β, IL-6 and IL-8) and the vascular endothelial growth factor (VEGF) by fiber-stimulated human mesothelial cells *in vitro* [63]. They showed that mesothelial cells secrete IL-1β in response to asbestos/erionite and that through an autocrine stimulation they undergo transformation [63]. In addition, the authors demonstrated that treatment of MM tumor-bearing SCID mice with IL-1R (Interleukin-1 receptor) antagonist (Anakinra) decreased the levels of IL-8 and VEGF in peritoneal lavage fluid, thus indicating that IL-1 has a key role in regulating the production of other cytokines, thus affecting the tumorigenesis of mesothelial cells [63]. A combination of IL-1β and TNF-α and erionite, or at least two cytokines together without erionite, for at least four months, induced transformation of the immortalized, non-tumorigenic human mesothelial cell line (MeT-5A) *in vitro* [67]. 

Accordingly, the release of cytokines driving inflammation represents a hallmark of exposure to asbestos. Mesothelial inflammatory processes were reported to occur both in animal models and in the lungs of patients exposed to asbestos. The production of different cytokines by mesothelial cells indicates the particular transcriptional aptitude of mesothelial cells [68]. A cytokine network is established in the serous membranes after mesothelial cell injury. Among the other cytokines, chemokines produced by mesothelial cells can recruit leukocytes [68] (Figure 1). Driscoll *et al.* showed that alveolar macrophages release TNF-α and IL-1 in rats exposed to crocidolite fibers [69]. In addition, crocidolite enhanced the production of mitochondrial-derived hydrogen peroxide which in turn contributes to crocidolite activation of NF-κB and increased MIP-2 (Macrophage Inflammatory protein-2) gene expression in rat alveolar Type II cells [70]. Recently, Acencio *et al.* performed an *in vitro* experiment to determine the acute inflammatory response of mesothelial cells damaged by asbestos fibers. They showed that mesothelial cells exposed to either crocidolite or chrysotile produced high levels of IL-6, IL-1β, MIP-2 and that these cytokines, when acting together with asbestos, increased cell death of pleural mesothelial cells [49]. Indeed, they showed that anti-IL-1β and anti-IL-6 antibodies significantly inhibited necrosis and apoptosis of mesothelial cells exposed to crocidolite [49]. High levels of cytokines, including transforming growth factor beta (TGF-β), IL-6, IL-1 and TNF-α were produced during MM development in an *in vivo* mouse model by the MM cells and/or tumor infiltrating leukocytes [71]. TNF-α, IL-6, TGF-β, and IL-10 have been shown to participate in cancer initiation and progression [72]. TGF-β counteracts proliferation and differentiation of different immune cells, thus inducing immunosuppression and favoring cancer cell growth [73]. IL-1β may confer a proliferative advantage to cancer cells through autocrine mechanisms [74]. The pro-inflammatory cytokines IL-1β, IL-6, IL-8, and VEGF promote tumor angiogenesis [75].

Fox *et al.* investigated the expression of CC and CXC chemokine genes I response to cytokines in MM and mesothelial cell cultures derived from two different mouse strains (BALB/*c* and CBA/CaH). They found that monocyte chemoattractant protein-1 (MCP-1)/JE, GRO-α/KC and RANTES were expressed in mouse MM and mesothelial cells, whereas MIP-1α and MIP-2 were infrequently expressed in these cell lines. MCP-1 was up-regulated in response to TNF-α and other cytokines [76]. MCP-1 and RANTES have been shown to induce cell growth and to act as monocyte attractants [77]. GRO-α/KC mRNA was overexpressed in cancer cells [76]. 

*In vivo* human studies were performed as well. The study of the RENAPE (French Network for Rare Peritoneal Malignancies) aimed to evaluate the intraperitoneal levels of IL-6, IL-8, IL-10, TNF-α, and sICAM (soluble intercellular adhesion molecule) in patients with pseudomyxoma peritonei and peritoneal mesothelioma. They found that cancer patients had significantly higher intraperitoneal cytokine levels than non-cancer patients. Cytokines peritoneal levels were significantly higher in peritoneal fluids compared with matched sera, thus indicating the cytokines production from either peritoneal cells or immune cells. In addition, they found a correlation between cytokine peritoneal levels and aggressiveness of peritoneal surface malignancies [78]. 

Comar *et al.,* employing Luminex Multiplex Panel Technology, measured the serum levels of a large panel of cytokines and growth factors from workers previously exposed to asbestos (Asb-workers). They found that interferon (IFN)-α, EOTAXIN, and RANTES were highly expressed in Asb-workers while IL-12(p40), IL-3, IL-1α, MCP-3, β-NGF (nerve growth factor), TNF-β, and RANTES were highly produced in MM patients [79]. 

Xu *et al.* found that the amount of CCL3 in the serum of healthy subjects potentially exposed to asbestos was significantly higher than for the control group. In addition, they observed that the pleural plaque, benign hydrothorax asbestosis, and lung cancer patients had serum CCL3 levels similar to that of healthy subjects potentially exposed to asbestos. They detected the CCL3 chemokine in the serum of nine of the 10 patients diagnosed with MM and three patients with MM showed very high CCL3 levels [80]. The elevated levels of CCL3 are very likely produced by macrophages chronically interacting with asbestos fibers [80]. 

### 4.3. Innate Immunity and Cytokines in the Development of MM: MM-Driven Immunoediting

The activation of an adaptive immune response and/or cell proliferation by inflammasome effectors is dependent on the cell type and tissue microenvironment [81]. The creation of a local cytokine-based microenvironment is employed by MM to avoid the specific immune response. IL-1β and IL-18 released by epithelial cells promote a Th2 response and recruitment of suppressive immune cells in the absence of IL-12 rather than activating Th1 and Th17 cells. In addition, the release of growth factors will favor angiogenesis and tumor invasiveness [81]. Indeed, IL-1β promotes carcinogenesis and induces the invasive potential of malignant cells by favoring the expression of matrix metalloproteinases, VEGF, chemokines, growth factors, and TGF-β in chronic inflamed tissue [82]. However, inflammasome’s activation in dendritic cells (DCs) and macrophages can bias Th1, Th17 immune response ability to reduce tumor growth in the presence of an appropriate microenvironment [81]. We recently demonstrated that macrophages and CD4+ T-cells were polarized by MM to produce IL-17, and that this cytokine exerts multiple tumor-supporting effects on both cell growth and invasiveness [83]. 

Many MM-derived factors can skew monocyte development through the recruitment of tumor-supporting cells, as reported by the presence of myeloid-derived suppressor cells (MDSCs) in murine models of MM. Employing a mouse model of transplanted diffuse MM, it was reported that MDSCs arise simultaneously with the recruitment of inflammatory cells in tumor foci. The presence of MDSCs came before the accumulation of macrophages and regulatory T lymphocytes which suppress T-cell function [84]. The cytokine profile three weeks after MM injection induced a tumor microenvironment that suppressed immune surveillance and antitumor immunity. At that stage, high expression levels of CXCL12, a chemotactic factor for MDSC, CCL9, and CXCL5, were observed [84]. Veltman *et al.* demonstrated that BALB/*c* mice carrying MM have PMN-MDSCs that induce immunosuppressive activity by releasing ROS via a cyclooxygenase-2 (COX-2)-dependent mechanism, which then induces T-cell immunosuppression [85]. The same authors inoculated mice with MM cells and treated them with celecoxib, a COX-2 inhibitor. They observed that treatment of tumor-bearing mice with the celecoxib prevented the local and systemic expansion of all MDSC subtypes [86]. However, a recent study by Yang *et al.* also reported that aspirin (a COX inhibitor) exerted a protective effect against MM growth through a COX-2-independent mechanism. In fact, the authors demonstrated that aspirin inhibited MM growth in a xenograft model by inhibiting the activities of HMGB1. The authors concluded that aspirin could be administered to people who were exposed to asbestos or erionite to prevent or delay MM development and progression [87]. 

Tumor-associated macrophages (TAMs) represent a major link between inflammation and cancer [88]. M1 macrophages have immunostimulatory Th1-activating properties while M2 cells have poor antigen-presenting capacity and suppress Th1 adaptive immunity [88]. Prostaglandin E2 (PGE2), TGF-β, IL-6, and IL-10 promote M2 macrophage polarization. Inhibition of the antitumor responses is achieved not only by the secretion of immunosuppressive cytokines but also by the selective recruitment of naive T-cells, trough CCL18, and of Th2 and Treg, through CCL17 and CCL22 [88]. The majority of TAMs in MM have the M2 phenotype. By retrospectively reviewing 667 tumor specimens of patients with MM it was found that, within the tumors, macrophages comprised 27% of the tumor area and had an immunosuppressive phenotype [89]. Hegmans *et al.* detected in pleural effusion of MM patients several cytokines involved in immune suppression and angiogenesis, including TGF-β. In addition, they demonstrated that human MM tissue contained a high number of Foxp3+ CD4+ CD25+ regulatory T-cells and when the CD25+ regulatory T-cells were depleted in an *in vivo* mouse model, mice survival increased [90]. The expression profile of cytokines and chemokines in mice transplanted with MM cells was consistent with M2-polarized cells [91]. They found elevated IL-10 and IL-10RA expression as well as expression of CXCL13, CCL22, CCL24, and their respective receptors [91]. In a recent report by Napolitano *et al.*, it was also observed that mice with germline BAP1 (BRCA1-associated protein-1) mutations (BAP1^+/−^ mice) exposed to low-dose asbestos fibers had alterations in the peritoneal inflammatory response. In fact, BAP1^+/−^ mice showed higher levels of pro-tumorigenic M2 macrophages and lower levels of M1 macrophages, cytokines (IL-6, leukemia inhibitory factor), and chemokines (MCP-1, keratinocyte-derived chemokine). Thus, these mice showed higher MM incidence after exposure to very low doses of asbestos, doses that rarely induced MM in wild-type mice. The authors suggested that patients with this mutation have an increased risk of developing MM, even after a minimal exposure of asbestos, due to alterations of the inflammatory response [92].

**Figure 2:**
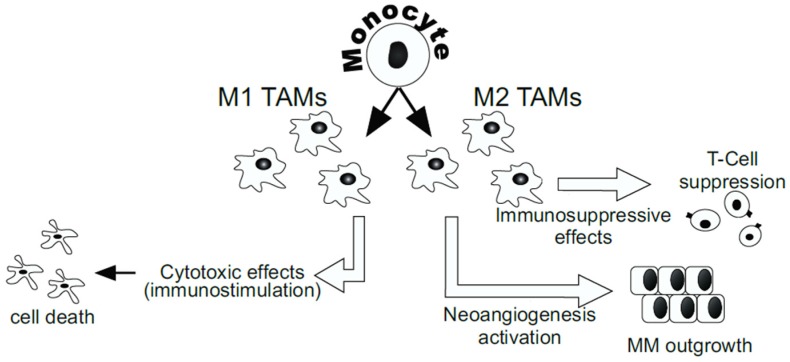
Role of the innate immunity in the development of MM. Tumor-associated macrophages (TAMs) represent a major link between inflammation and cancer. The majority of TAMs in MM have the M2 phenotype. M2 TAMs have poor antigen-presenting capacity, suppress T-cells adaptive immunity, and support MM growth.

Asbestos induces partially functional decreases in T helper (Th) cells, natural killer (NK) cells, and cytotoxic T lymphocytes (CTLs) in patients with MM [93]. To elucidate the antitumor immune interference of asbestos caused to CD4+ T-cells, Maeda *et al.* established an *in vitro* T-cell model of long-term and low-level exposure to chrysotile asbestos from a human adult T-cell leukemia virus-1-immortalized human polyclonal CD4+ T-cell line (MT-2). They observed a decreased expression of CXCR3, IFN-γ, and CXCL10/IP10 in the MT-2 cell line, thus suggesting that exposure to asbestos may impair the antitumor immune responses [94]. They also found that chrysotile asbestos reduces the chemokine receptor CXCR3 expression in human peripheral CD4+ T-cells, thus suggesting that immune response might be impaired in patients with asbestos-related disease because the low expression of CXCR3 might reduce chemotaxis [95]. In addition, in a recent report, the same authors showed that an asbestos-induced apoptosis-resistant subline (MT-2Rst), which was established from a human adult T-cell leukemia virus-immortalized T-cell line (MT-2Org) by continuous exposure to asbestos chrysotile-B, produced high levels of TGF-β1 through phosphorylation of p38 MAPK, and acquire resistance to inhibition of cell growth by TGF-β1 [96]. It was observed that asbestos can trigger a cascade of biological events including the increase of IL-10 expression and Bcl-2 overexpression in human T-cell leukemia virus-immortalized T-cell line and that CD4+ T lymphocytes from MM patients had significant up-regulation of Bcl-2 expression thus affecting their survival. The Bcl-2 up-regulation might affect the Treg population thus contributing to immunosuppression in cancer patients [97] (Figure 2).

## 5. Effects of Polyphenols in MM

### 5.1. Effects of Polyphenols on ROS in MM

Epidemiological studies indicate the existence of an inverse correlation between the consumption of polyphenols and the incidence of various chronic diseases and cancer. In fact, polyphenols possess anti-oxidant activities and thus are able to protect cells from oxidative stress, providing an anti-inflammatory effect [98,99,100]. For instance, flavonoids are able to scavenge ROS generated by neutrophils and macrophages and to impair ROS production by inhibiting NADPH oxidase, xanthine oxidase, and myeloperoxidase [27,29,30]. In addition, polyphenols modulate the activity of ROS-generating enzymes, such as COX and lipoxygenase (LOX) [30,101,102]. Furthermore, polyphenols inhibit NO production from activated macrophages [103,104] and also inducible nitric oxide synthase (iNOS) protein and its mRNA expression [105].

However, polyphenols also possess a pro-oxidant activity, depending on their concentration and chemical structure, cell type, or experimental conditions (pH, redox stress) [106,107]. The pro-oxidant effect of polyphenols is important in cancer cells, since this effect leads to oxidative breaking of DNA, inhibition of cell growth and apoptosis [14]. In fact, in the last few years the use of pro-oxidants against cancer is an emerging topic of research, since it has been observed that ROS contribute to the cytotoxic activity of some chemotherapeutics and that cancer cells are more susceptible to ROS than normal cells [107]. 

As for MM, asbestos produces ROS and RNS, that act as second messengers to drive initiation and progression of MM-carcinogenesis, through genetic alterations, activation of the survival pathways, stimulation of matrix metalloproteinases (MMP), and angiogenic signaling. Furthermore, ROS mediate extrinsic and intrinsic pathways of apoptosis, necrosis, and autophagy, thus ROS production is also used as a therapy for MM, to limit tumor growth [51]. In this way, polyphenols, which also possess pro- and anti-oxidant properties, are a promising tool to treat MM.

Several studies have explored the ability of different polyphenols as pro-oxidant agents in MM. It has been demonstrated that curcumin (40 μM) increased ROS production in HMESO cells *in vitro*, leading to caspase-1 activation and pyroptotic cell death of MM cells [108]. 

Satoh *et al.* demonstrated that the flavan-3-ol epigallocatechin-3-gallate (EGCG) induced ROS production and impaired the mitochondrial membrane potential and these effects were responsible for the induction of apoptosis in MM cells *in vitro*. In fact, the treatment of MM cells with ROS scavengers, such as tempol and catalase, inhibited the apoptosis induced by EGCG [109]. A similar effect was reported by Ranzato *et al.* They demonstrated that EGCG induced both apoptotic and necrotic cell death in MM cells. In particular, it has been shown that EGCG had a pro-oxidant effect and induced cell death by the release of H_2_O_2_ outside of cells [110]. Similarly, a recent study showed that EGCG, when added to culture medium, induced H_2_O_2_ formation and decreased proliferation both in MM cells and MET5A cells (normal cells). Due to EGCG instability that causes H_2_O_2_ formation in culture medium, ECGC was added to cells in presence of catalase (CAT) and exogenous superoxide dismutase (SOD). In this way, EGCG decreased cell proliferation only in MM cells and induced mitochondrial apoptosis [111].

The increased levels of ROS induce the nuclear translocation and activation of Nrf2 (nuclear factor E2-related factor 2) in MM. Normally, Nrf2 is sequestered in cytosol by its inhibitor Keap-1 (Kelch-like ECH-associated protein 1); when MM arises, the ROS levels increase and one or multiple cysteines bind to Keap-1, which undergoes a conformational change releasing Nrf2. Next, Nrf2 translocates to the nucleus and activates the transcription of downstream genes, as HO-1. The high levels of Nrf2 create an anti-oxidant environment which is resistant to MM-therapy. It has been demonstrated that the combined treatment of clofarabine and resveratrol inhibited the Nrf2 pathway by reducing nuclear localization of Nrf2 and by decreasing Nrf2 and HO-1 protein levels *in vitro*. Lee *et al.* hypothesized that resveratrol with clofarabine decreased the chemoresistance of MM, modulating the levels of proteins activated by ROS, as Nrf2 [112]. In addition, the same authors demonstrated that the combined treatment of clofarabine and resveratrol increased the nuclear expression of phospho-p53. Hence, p53 induced the expression of pro-apoptotic proteins, as Bax, Puma, and Noxa [113]. Faraonio *et al.* indicated the possibility of crosstalk between p53 and Nrf2. p53 could prevent the generation of an anti-oxidant environment counteracting the effect of Nrf2 and inducing apoptosis [114]. 

A recent study by Pietrofesa *et al.* reported the *in vitro* ability of LGM2605 (a synthetic lignan secoisolariciresinol diglucoside) to reduce asbestos-induced cytotoxicity and ROS generation and to induce phase II anti-oxidant enzymes stimulated by Nrf2 (HO-1 and Nqo1) in murine peritoneal macrophages. LGM2605 acted as a direct free radical scavenger and anti-oxidant in a dose-dependent manner. They hypothesized the possible use of this synthetic lignan as a chemopreventive agent in the development of asbestos-induced MM [115]. 

Kostyuk *et al.* have conducted several studies on the efficacy of different polyphenols in preventing asbestos-induced injury of peritoneal macrophages and red blood cells. They demonstrated that quercetin and rutin were able to reduce peritoneal macrophages injury caused by asbestos and to scavenge ROS. They suggested that quercetin and rutin could be promising drug candidates for a prophylactic asbestos-induced disease [116]. Similarly, in another study they explored the efficacy of the main polyphenolic constituents of green tea extract, (−)-epicatechin gallate (ECG) and (−)-epigallocatechin gallate (EGCG). They observed that ECG and EGCG had a protective effect against chrysotile and crocidolite-induced cell injuries in peritoneal macrophages, and this effect was attributed to the scavenger properties towards the superoxide anion and the ability of polyphenols to chelate iron ions [117]. They also concluded in a comparative study that the protective effect increased in the following series: rutin < dihydroquercetin < quercetin < ECG < EGCG [118].

Effects of polyphenols on ROS in MM are summarized in Table 1. 

### 5.2. Effects of Polyphenols on Mediators of Inflammation in MM

Inflammation plays a critical role in the process of carcinogenesis by regulating the different stages of initiation, promotion, progression, and metastasis, and also the responses to therapies [119]. In this regard, it has been observed that the tumor microenvironment is infiltrated by innate and adaptive immune cells, such as macrophages, neutrophils, mast cells, myeloid-derived suppressor cells, dendritic cells, NKcells, and T and B lymphocytes that communicate to each other through the production of cytokines [119]. 

Polyphenols possess the ability to directly modulate innate and also adaptive immune cells that infiltrate the tumor. In fact, it has been demonstrated that different polyphenols, such as genistein, EGCG, curcumin, and resveratrol, are able to modulate these immune cells to enhance an antitumor response or to suppress the immune escape of tumors [14].

In addition, it has been demonstrated that polyphenols possess the ability to control the inflammatory process by inhibiting the secretion of pro-inflammatory cytokines (IL-1β, IL-2, IL-6, IFN-γ, TNF-α) and chemokines [20]. The inhibition of the production of these cytokines also led to inhibition of ROS, since cytokines trigger ROS production [120]. Several studies have reported this ability of polyphenols. For instance, curcumin and different flavonoids, such as flavones, EGCG, and flavonols, are able to inhibit the secretion of TNF-α, IL-6, IL-1β, IL-8, and IFN-γ from various cell types [121,122,123,124,125,126,127,128,129]. 

By the activation of different transcription factors, such as NF-κB, AP-1, STAT-3, SMAD, and caspases, cytokines can promote or inhibit tumor progression [119]. It has been demonstrated that polyphenols, such as resveratrol, flavones, flavonols, EGCG, anthocyanins, isoflavones, and curcumin, are able to modulate NF-κB [130,131,132,133,134,135,136,137,138]. Curcumin, resveratrol, and EGCG also inhibit STAT-3 activation [139,140,141,142].

Inflammation, and thus the production of inflammasome, has an essential role in the development of MM [143]. As previously described, the active inflammasome induces the activation of caspase-1 and mature form of pro-inflammatory cytokines IL-1β and IL-18 and thus the inflammatory cell death pyroptosis [144]. 

In this regard, the anti-inflammatory effect of polyphenols, by regulating innate and adaptive immunity through the modulation of different cytokines, chemokines, and transcription factors could be a promising strategy to contrast development of this type of cancer [14]. 

Miller *et al.* has observed that curcumin was able to kill MM cells *via* pyroptosis without the classical inflammasome-related cytokines, IL-1β and IL-18. They observed that curcumin increased the concentration of caspase-1 but did not increase IL-1β and IL-18 expression. Furthermore, they observed a higher concentration of pro-IL-1β, indicating a block of the maturation of cytokine. Curcumin treatment increased the expression of NLRP3, which alone induces a decreased NF-κB expression. Curcumin reduced the inflammasome-related gene expression, NF-κB, TLR and IL-1 pathway. In addition, curcumin down-regulated the expression of MYD88, NLRC4, and TXNIP and up-regulated HSP90AA1 (heat shock protein 90 kDa alpha class A member 1), IL-12, IL-6. Hence, curcumin has an anti-inflammatory effect on MM cells by blocking cytokine processing of IL-1β and genes involved in the NF-κB pathway [108].

Wang *et al.* demonstrated that curcumin also suppressed MM cell growth *in vitro* and *in vivo* (oral administration) and enhanced the efficacy of cisplatin. In particular, curcumin inhibited cell growth through activation of p38 kinase, caspases 9 and 3, increased pro-apoptotic protein Bax levels, stimulated PARP cleavage, and induced apoptosis. In addition, curcumin stimulated expression of novel transducers of cell growth suppression, such as CARP-1, XAF1, and SULF1 proteins [145].

It has been shown that the activated NF-κB and high levels of the activated phosphorylated STAT-3 are present in MM. Cioce *et al.* showed that butein (3,4,2′,4′-tetrahydroxychalcone), a natural inhibitor of NF-κB and STAT-3, inhibits the migration of MM cells and strongly affects the clonogenicity of MM cells *in vitro* by inhibiting the phosphorylation of STAT-3, the nuclear localization of NF-κB and the interaction of NF-κB and phospho-STAT-3. Different genes involved in cancer progression of pro-angiogenic cytokines (VEGF) and of IL-6 and IL-8 were also down-regulated. Furthermore, they showed that butein was able to severely affect tumor engraftment and to potentiate the anticancer effects of pemetrexed in mouse xenograft models *in vivo*. Intraperitoneal treatment with butein was safe, since butein does not significantly affect the viability of human untransformed mesothelial cells *in vitro* or the survival of tumor-free mice *in vivo* [146].

The activation of STAT-3 is associated to PIAS-3 expression levels in MM cell lines. PIAS-3 specifically interacts with phospho-STAT-3 and decreases the STAT-3 DNA-binding capacity and transcriptional activity. The overexpression of PIAS-3 can inhibit STAT-3 transcriptional activity and induces apoptosis *in vitro* [147]. Dabir *et al.* demonstrated that an inverse correlation between PIAS-3 and STAT-3 is present in MM cells. In fact, they showed that high levels of phospho-STAT-3 and low levels of PIAS-3 are present. Furthermore, they observed that treatment with curcumin (1.0 µM) was able to increase PIAS-3 levels and thereby decreased STAT-3 phosphorylation and cell viability in MM cells [148]. 

Flaxseed lignans, enriched in secoisolariciresinol diglucoside (SDG), have been investigated for the prevention of asbestos-induced peritoneal inflammation in a mouse model of accelerated MM development that recapitulates many of the molecular, genetic, and cell-signaling features of human MM after asbestos injection. Mice were supplemented with a diet containing lignans seven days before an intraperitoneal injection of crocidolite asbestos and three days after asbestos exposure; they were evaluated for abdominal inflammation, pro-inflammatory/pro-fibrogenic cytokine release, WBC gene expression changes, and oxidative and nitrosative stress in peritoneal lavage fluid. The results showed that dietary lignan administration diminished acute inflammation by decreasing the number of WBCs and the release of IL-1β, IL-6, HMGB1, and TNF-α pro-inflammatory cytokines and pro-fibrogenic active TGF-β1. Furthermore, lignan acted as an anti-oxidant by decreasing mRNA levels of inducible nitric oxide synthase, and thus nitrosative and oxidative stress, and by increasing the expression of the Nrf2-regulated anti-oxidant enzymes, HO-1, Nqo1 and Gstm1 [42]. 

In a preliminary study, Martinotti *et al.* demonstrated that the combined treatment with EGCG, ascorbate, and gemcitabine (AND) synergistically affected the viability of MM cells [149]. Next, the same authors showed that AND treatment increased DAPK2 (Death-Associated Protein Kinase 2), a calcium- and calmodulin-dependent regulator of apoptosis and tumor suppressor, and TNSFR11B expression. The TNSFR11B gene encodes a cytokine receptor belonging to the TNF receptor family, called osteoprotegerin (OPG). OPG acts as receptor for RANK ligand, inhibiting RANK-dependent activation of NF-κB. Furthermore, they observed a decreased expression of TNFAIP3 (tumor necrosis factor-α-induced protein 3), an inhibitor of NF-κB activation and TNF-mediated apoptosis, typically up-regulated in inflammation and in tumors. In this study, they found a down-regulated TNFAIP3 expression because AND treatment decreased p65 subunit of NF-κB. Hence, the combined treatment induced a non-inflammatory apoptosis [150]. 

The transcription factor Specificity protein 1 (Sp1) is highly expressed in different cancers and is associated with poor prognosis. Sp1 modulates the expression of oncogenes and tumor suppressors, as well as genes involved in proliferation, differentiation, the DNA damage response, apoptosis, senescence, and angiogenesis and it is also implicated in inflammation and genomic instability [151].

Lee *et al.* showed that resveratrol decreased the Sp1 expression and down-regulated Sp1-dependent gene expression in MM. They observed a decreased tumor volume and an increased number of caspase-3-positive cells after intraperitoneal treatment with resveratrol [152]. In another study, it has been demonstrated that the combined treatment of clofarabine and resveratrol decreased levels of Sp1, p-Akt, c-Met, cyclin D1, and p21 [153].

Similarly, Chae *et al.* found that 20–80 µM quercetin suppressed the Sp1 expression and modulated the target genes, as cyclin D1, Mcl-1 (myeloid cell leukemia), and survivin in MM. Furthermore, quercetin induced apoptosis through the Bid, caspase-3, and PARP cleavage, the up-regulation of Bax, and down-regulation of Bcl-xL in MSTO-211H cells [154].

In another study, the same authors focused on the anticancer effects of honokiol (HNK), a pharmacologically active component found in the traditional Chinese medicinal herb, *Magnolia* species. It has been observed that HNK inhibited MM cell growth, down-regulated Sp1 expression and Sp1 target transcription factors, including cyclin D1, Mcl-1, and survivin, and induced the apoptosis by increasing Bax, reducing Bid and Bcl-xL and activating caspase-3 and PARP [155].

Kim *et al.* found that licochalcone A (LCA), a natural product derived from the Glycyrrhiza inflata, regulated the cell growth and down-regulated the Sp1 expression in MSTO-211H and H28 cell lines. Furthermore, LCA down-regulated the expression of Sp1 downstream genes, as cyclin D1, Mcl1 and survivin. Like quercetin and honokiol, LCA increased Bax and decreased Bcl-2 expression, inducing the mitochondrial apoptotic pathway [156].

Lee *et al.* demonstrated that hesperidin, a flavanone presents in citrus fruits, inhibited the cell growth and down-regulated the SP1 expression in MSTO-211H cells. Hesperidin significantly suppressed mRNA and protein levels of Sp1 and regulated the expression of p27, p21, cyclin D1, Mcl-1, and survivin. Furthermore, hesperidin induced the apoptosis pathway through cleavages of Bid, caspase-3, and PARP, and up-regulation of Bax and down-regulation of Bcl-xL [157]. Similarly, the same authors showed that cafestol and kahweol, two diterpenes present in the typical bean of *Coffea Arabica,* induced apoptosis and suppressed the Sp1 protein levels in MSTO-211H cells. These compounds modulated the expression of genes regulated by Sp1, including cyclin D1, Mcl-1, and survivin. Furthermore, the cafestol treatment induced the cleavage of Bid, caspase-3, and PARP, and the kahweol treatment up-regulated Bax and down-regulated Bcl-xL [158].

The effect of a novel mixture containing lysine, proline, ascorbic acid, and green tea extract has been investigated by Roomi *et al.* in MM cell line MSTO-211H. They demonstrated that this mixture was able to inhibit MMP secretion and invasion and thus is a promising candidate for therapeutic use in the treatment of MM [159].

Effects of polyphenols on mediators of inflammation in MM are summarized in Table 2.

## 6. Conclusions

The immune system, and in particular inflammation, has an essential role in the development of MM. A long-lasting inflammatory response orchestrated by mesothelial cells contributes to the initiation, promotion, and progression of mesothelial cells into MM. Polyphenols possess important anti-inflammatory properties by regulating innate and adaptive immunity through the modulation of different mediators of inflammation and also by acting as an immune surveillance mechanism against cancers through the regulation of apoptosis. Furthermore, polyphenols possess a pro-oxidant activity, which could be used against cancer. In fact, in the last few years the use of ROS-generating agents against cancer is an emerging strategy to kill cancer cells, since it has been observed that ROS contribute to the cytotoxic activity of some chemotherapeutics and that cancer cells are more susceptible to ROS than normal cells.

Accordingly, the local or systemic administration of polyphenols might reduce the production of pro-inflammatory molecules by targeting signal transduction pathways or ROS and RNS in order to prevent MM. On the other hand, the administration of polyphenols might also induce MM cell death to limit tumor growth. 

Furthermore, MM is a tumor arising from the mesothelial cell linings of the serous membranes, thus the local administration of polyphenols in the serous cavity might be a better strategy to treat MM, because in this way polyphenols could bypass biotransformation and could reach an effective dose directly available at the site of tumor. 

Thus the use of polyphenols might represent a promising strategy to contrast the development and/or to prevent MM.

## Figures and Tables

**Table 1:** Effects of polyphenols on ROS production and scavenging in MM.

Polyphenols	Cell Type	Effects on ROS	Ref.
Curcumin	H-MESO cells	↑ ROS	[108]
		↑ Caspase-1	
		↑ Pyroptotic cell death	
EGCG	ACC-meso 1, Y-meso 8A, EHMES-10, EHMES-1, MSTO-211H, REN, MM98, BR95, E198 cells	↑ ROS	[109,110,111]
		↑ H_2_O_2_ outside of cells	
		↑ Apoptosis and necrosis	
		↓ Cell proliferation	
Resveratrol (+Clofarabine)	MSTO-211H cells	↓ Nrf2 pathway	[112,113]
		↑ p53 phosphorylation	
		↑ Pro-apoptotic proteins	
LGM2605 (a synthetic lignan)	Murine peritoneal macrophages	↓ ROS	[115]
		↓ Cytotoxicity	
		↑ Phase II anti-oxidant enzymes	
Quercetin + Rutin	Peritoneal macrophages of Wistar rats	↓ ROS	[116]
		↓ Peritoneal macrophages injury by asbestos	
EGCG + ECG	Peritoneal macrophages of Wistar rats	↓ ROS	[117]
		↓ Peritoneal macrophages injury by asbestos	

↓: decrease; ↑: increase.

**Table 2:** Effects of polyphenols on production of mediators of inflammation in MM.

Polyphenols	Cell Type or Animal Model	Effects on Inflammation	Ref.
Curcumin	H-MESO, NCI-2052, NCI-H2452, MSTO-211H, and NCI-H28 cells	↑ Caspase-1	[108,148]
		↑ pro-IL-1β and block of maturation of IL-1 β	
		↑ NLRP3	
		↓ NF-κB, TRL, and IL-1 pathways	
		↑ PIAS-3	
		↓ p-STAT-3	
Butein	MSTO-211H, NCI-H28, NCI-H2052	↓ NF-κB, p-STAT-3	[146]
		↓VEGF	
		↓ IL-6, IL-8	
Flaxseed Lignans	MM-prone Nf2^+/mut^ mice	↓ IL-1β, IL-6, HMGB1, TNF-α, TGF-β1	[42]
		↑ Nrf2-regulated anti-oxidant enzymes	
EGCG + Ascorbate + Gemcitabine (AND)	REN cells	↑ DAPK2	[150]
		↑ TNSFR11B	
		↓ TNFAIP3	
		↓ NF-κB pathway	
Resveratrol	MSTO-211H cells	↓ Sp1, p21, p27, cyclin D1, Mcl-1	[152]
		↓ survivin	
		↑ Apoptosis	
Resveratrol + Clofarabine	MSTO-211H cells	↓ Sp1, p-Akt	[153]
		↓ c-Met, cyclin D1, p21	
Quercetin	MSTO-211H cells	↓ Sp1, cyclin D1, Mcl-1, survivin	[154]
		↑ Apoptosis	
Honokiol	MSTO-211H cells	↓ Sp1	[155]
		↓ cyclin D1, Mcl-1, survivin	
		↑ Apoptosis	
Licochalcone A	MSTO-211H and H28 cells	↓ Sp1	[156]
		↓ cyclin D1, Mcl-1, survivin	
		↑ Apoptosis	
Hesperidin	MSTO-211H cells	↓ Sp1	[157]
		↓ p27, p21, cyclin D1, Mcl-1, survivin	
		↑ Apoptosis	
Cafestol and kahweol	MSTO-211H cells	↓ Sp1, cyclin D1, Mcl-1, survivin	[158]
		↑ Apoptosis	

**↓**: decrease; **↑**: increase.

## References

[1] Carbone M., Ly B.H., Dodson R.F., Pagano I., Morris P.T., Dogan U.A., Gazdar A.F., Pass H.I., Yang H. (2012). Malignant mesothelioma: Facts, myths, and hypotheses. J. Cell Physiol..

[2] Yang H., Rivera Z., Jube S., Nasu M., Bertino P., Goparaju C., Franzoso G., Lotze M.T., Krausz T., Pass H.I. (2010). Programmed necrosis induced by asbestos in human mesothelial cells causes high-mobility group box 1 protein release and resultant inflammation. Proc. Nat. Acad. Sci. USA.

[3] Jube S., Rivera Z.S., Bianchi M.E., Powers A., Wang E., Pagano I., Pass H.I., Gaudino G., Carbone M., Yang H. (2012). Cancer cell secretion of the DAMP protein HMGB1 supports progression in malignant mesothelioma. Cancer Res..

[4] Napolitano A., Antoine D.J., Pellegrini L., Baumann F., Pagano I., Pastorino S., Goparaju C.M., Prokrym K., Canino C., Pass H.I. (2016). HMGB1 and its hyperacetylated isoform are sensitive and specific serum biomarkers to detect asbestos exposure and to identify mesothelioma patients. Clin. Cancer Res..

[5] Remon J., Lianes P., Martinez S., Velasco M., Querol R., Zanui M. (2013). Malignant mesothelioma: New insights into a rare disease. Cancer Treat. Rev..

[6] Faig J., Howard S., Levine E.A., Casselman G., Hesdorffer M., Ohar J.A. (2015). Changing Pattern in Malignant Mesothelioma Survival. Transl. Oncol..

[7] Testa J.R., Cheung M., Pei J., Below J.E., Tan Y., Sementino E., Cox N.J., Dogan A.U., Pass H.I., Trusa S. (2011). Germline BAP1 mutations predispose to malignant mesothelioma. Nat. Genet..

[8] Astoul P., Roca E., Galateau-Salle F., Scherpereel A. (2012). Malignant pleural mesothelioma: From the bench to the bedside. Respiration.

[9] Izzi V., Masuelli L., Tresoldi I., Foti C., Modesti A., Bei R. (2012). Immunity and malignant mesothelioma: From mesothelial cell damage to tumor development and immune response-based therapies. Cancer Lett..

[10] Menges C.W., Chen Y., Mossman B.T., Chernoff J., Yeung A.T., Testa J.R. (2010). A phosphotyrosine proteomic screen identifies multiple tyrosine kinase signaling pathways aberrantly activated in malignant mesothelioma. Genes Cancer.

[11] Albonici L., Palumbo C., Manzari V., Belli C. (2012). Role of inflammation and angiogenic growth factors in malignant mesothelioma. Malignant Mesothelioma.

[12] Bajaj G., Yeo Y. (2010). Drug delivery systems for intraperitoneal therapy. Pharm. Res..

[13] Benvenuto M., Fantini M., Masuelli L., de Smaele E., Zazzeroni F., Tresoldi I., Calabrese G., Galvano F., Modesti A., Bei R. (2013). Inhibition of ErbB receptors, Hedgehog and NF-kappaB signaling by polyphenols in cancer. Front. Biosci..

[14] Ghiringhelli F., Rebe C., Hichami A., Delmas D. (2012). Immunomodulation and anti-inflammatory roles of polyphenols as anticancer agents. Anticancer Agents Med. Chem..

[15] Gonzáles R., Ballester I., López-Posadas R., Suárez M.D., Zarzuelo A., Martínez-Augustin O., de Medina F.S. (2011). Effects of flavonoids and other polyphenols on inflammation. Crit. Rev. Food Sci. Nutr..

[16] Santangelo C., Vari R., Scazzocchio B., di Benedetto R., Filesi C., Masella R. (2007). Polyphenols, intracellular signalling and inflammation. Ann. Ist. Super. Sanità.

[17] Cuevas A., Saavedra N., Salazar L.A., Abdalla D.S. (2013). Modulation of immune function by polyphenols: Possible contribution of epigenetic factors. Nutrients.

[18] Manach C., Scalbert A., Morand C., Rémésy C., Jiménez L. (2004). Polyphenols: Food sources and bioavailability. Am. J. Clin. Nutr..

[19] Scalbert A., Manach C., Morand C., Rémésy C., Jiménez L. (2005). Dietary polyphenols and the prevention of diseases. Crit. Rev. Food Sci. Nutr..

[20] Marzocchella L., Fantini M., Benvenuto M., Masuelli L., Tresoldi I., Modest A., Bei R. (2011). Dietary flavonoids: Molecular mechanisms of action as anti- inflammatory agents. Recent Patent Inflamm. Allergy Drug Disc..

[21] Crozier A., Jaganath I.B., Clifford M.N. (2009). Dietary phenolics: Chemistry, bioavailability and effects on health. Nat. Prod. Rep..

[22] Fantini M., Benvenuto M., Masuelli L., Frajese G.V., Tresoldi I., Modesti A., Bei R. (2015). *In vitro* and *in vivo* antitumoral effects of combinations of polyphenols, or polyphenols and anticancer drugs: Perspectives on cancer treatment. Int. J. Mol. Sci..

[23] Bei R., Masuelli L., Turriziani M., Li Volti G., Malaguarnera M., Galvano F. (2009). Impaired expression and function of signaling pathway enzymes by anthocyanins: Role on cancer prevention and progression. Curr. Enzym. Inhib..

[24] Tomás-Barberán F.A., Clifford M.N. (2000). Flavanones, chalcones and dihydrochalcones-nature, occurrence and dietary burden. J. Sci. Food Agric..

[25] Cassidy A., Hanley B., Lamuela-Raventos R.M. (2000). Isoflavones, lignans and stilbenes-origins, metabolism and potential importance to human health. J. Sci. Food Agric..

[26] Prasad S., Tyagi A.K., Aggarwal B.B. (2014). Recent developments in delivery, bioavailability, absorption and metabolism of curcumin: The golden pigment from golden spice. Cancer Res. Treat..

[27] García-Lafuente A., Guillamón E., Villares A., Rostagno M.A., Martínez J.A. (2009). Flavonoids as anti-inflammatory agents: Implications in cancer and cardiovascular disease. Inflamm. Res..

[28] Izzi V., Masuelli L., Tresoldi I., Sacchetti P., Modesti A., Galvano F., Bei R. (2012). The effects of dietary flavonoids on the regulation of redox inflammatory networks. Front. Biosci..

[29] Edwards S.W. (1996). The O-2 Generating NADPH Oxidase of Phagocytes: Structure and Methods of Detection. Methods.

[30] Cotelle N. (2001). Role of flavonoids in oxidative stress. Curr. Top. Med. Chem..

[31] Manach C., Williamson G., Morand C., Scalbert A., Rémésy C. (2005). Bioavailability and bioefficacy of polyphenols in humans. I. Review of 97 bioavailability studies. Am. J. Clin. Nutr..

[32] Ginsburg I., Kohen R., Koren E. (2011). Microbial and host cells acquire enhanced oxidant-scavenging abilities by binding polyphenols. Arch. Biochem. Biophys..

[33] Bohn T. (2014). Dietary factors affecting polyphenol bioavailability. Nutr. Rev..

[34] Masuelli L., Fantini M., Benvenuto M., Sacchetti P., Giganti M.G., Tresoldi I., Lido P., Lista F., Cavallo F., Nanni P. (2014). Intratumoral delivery of recombinant vaccinia virus encoding for ErbB2/Neu inhibits the growth of salivary gland carcinoma cells. J. Transl. Med..

[35] Masuelli L., Marzocchella L., Focaccetti C., Lista F., Nardi A., Scardino A., Mattei M., Turriziani M., Modesti M., Forni G. (2010). Local delivery of recombinant vaccinia virus encoding for neu counteracts growth of mammary tumors more efficiently than systemic delivery in neu transgenic mice. Cancer Immunol. Immunother..

[36] Galanis E., Russell S. (2001). Cancer gene therapy clinical trials: Lessons for the future. Br. J. Cancer.

[37] Forsyth P., Roldán G., George D., Wallace C., Palmer C.A., Morris D., Cairncross G., Matthews M.V., Markert J., Gillespie Y. (2008). A phase I trial of intratumoral administration of reovirus in patients with histologically confirmed recurrent malignant gliomas. Mol. Ther..

[38] Roberts N.J., Zhang L., Janku F., Collins A., Bai R.Y., Staedtke V., Rusk A.W., Tung D., Miller M., Roix J. (2014). Intratumoral injection of Clostridium novyi-NT spores induces antitumor responses. Sci. Transl. Med..

[39] Fujiwara S., Wada H., Miyata H., Kawada J., Kawabata R., Nishikawa H., Gnjatic S., Sedrak C., Sato E., Nakamura Y. (2012). Clinical trial of the intratumoral administration of labeled DC combined with systemic chemotherapy for esophageal cancer. J. Immunother..

[40] Masuelli L., Pantanella F., la Regina G., Benvenuto M., Fantini M., Mattera R., Di Stefano E., Mattei M., Silvestri R., Schippa S. (2016). Violacein, an indole-derived purple-colored natural pigment produced by *Janthinobacterium lividum*, inhibits the growth of head and neck carcinoma cell lines both *in vitro* and *in vivo*. Tumour Biol..

[41] Price B., Ware A. (2009). Time trend of mesothelioma incidence in the United States and projection of future cases: An update based on SEER data for 1973 through 2005. Crit. Rev. Toxicol..

[42] Pietrofesa R.A., Velalopoulou A., Arguiri E., Menges C.W., Testa J.R., Hwang W.T., Albelda S.M., Christofidou-Solomidou M. (2016). Flaxseed lignans enriched in secoisolariciresinol diglucoside prevent acute asbestos-induced peritoneal inflammation in mice. Carcinogenesis.

[43] Chew S.H., Toyokuni S. (2015). Malignant mesothelioma as an oxidative stress-induced cancer: An update. Free Radic. Biol. Med..

[44] Otsuki T., Maeda M., Murakami S., Hayashi H., Miura Y., Kusaka M., Nakano T., Fukuoka K., Kishimoto T., Hyodoh F. (2007). Immunological effects of silica and asbestos. Cell. Mol. Immunol..

[45] Miserocchi G., Sancini G., Mantegazza F., Chiappino G. (2008). Translocation pathways for inhaled asbestos fibers. Environ. Health.

[46] Tunesi S., Ferrante D., Mirabelli D., Andorno S., Betti M., Fiorito G., Guarrera S., Casalone E., Neri M., Ugolini D. (2015). Gene-asbestos interaction in malignant pleural mesothelioma susceptibility. Carcinogenesis.

[47] Jantz M.A., Antony V.B. (2008). Pathophysiology of the pleura. Respiration.

[48] Carbone M., Bedrossian C.W. (2006). The pathogenesis of mesothelioma. Semin. Diagn. Pathol..

[49] Acencio M.M., Soares B., Marchi E., Silva C.S., Teixeira L.R., Broaddus V.C. (2015). Inflammatory cytokines contribute to asbestos-induced injury of mesothelial cells. Lung.

[50] Carbone M., Yang H. (2012). Molecular pathways: Targeting mechanisms of asbestos and erionite carcinogenesis in mesothelioma. Clin. Cancer Res..

[51] Benedetti S., Nuvoli B., Catalani S., Galati R. (2015). Reactive oxygen species a double-edged sword for mesothelioma. Oncotarget.

[52] Kamp D.W., Weitzman S.A. (1999). The molecular basis of asbestos induced lung injury. Thorax.

[53] Kinnula V.L., Aalto K., Raivio K.O., Walles S., Linnainmaa K. (1994). Cytotoxicity of oxidants and asbestos fibers in cultured human mesothelial cells. Free Radic. Biol. Med..

[54] Kinnula V.L., Raivio K.O., Linnainmaa K., Ekman A., Klockars M. (1995). Neutrophil and asbestos fiber-induced cytotoxicity in cultured human mesothelial and bronchial epithelial cells. Free Radic. Biol. Med..

[55] Moslen M.T. (1994). Reactive oxygen species in normal physiology, cell injury and phagocytosis. Adv. Exp. Med. Biol..

[56] Hansen K., Mossman B.T. (1987). Generation of superoxide (O2-.) from alveolar macrophages exposed to asbestiform and nonfibrous particles. Cancer Res..

[57] Shukla A., Gulumian M., Hei T.K., Kamp D., Rahman Q., Mossman B.T. (2003). Multiple roles of oxidants in the pathogenesis of asbestos-induced diseases. Free Radic. Biol. Med..

[58] Funahashi S., Okazaki Y., Ito D., Asakawa A., Nagai H., Tajima M., Toyokuni S. (2015). Asbestos and multi-walled carbon nanotubes generate distinct oxidative responses in inflammatory cells. J. Clin. Biochem. Nutr..

[59] Tanaka S., Choe N., Hemenway D.R., Zhu S., Matalon S., Kagan E. (1998). Asbestos inhalation induces reactive nitrogen species and nitrotyrosine formation in the lungs and pleura of the rat. J. Clin. Investig..

[60] Soini Y., Kahlos K., Puhakka A., Lakari E., Säily M., Pääkkö P., Kinnula V. (2000). Expression of inducible nitric oxide synthase in healthy pleura and in malignant mesothelioma. Br. J. Cancer.

[61] Reuter S., Gupta S.C., Chaturvedi M.M., Aggarwal B.B. (2010). Oxidative stress, inflammation, and cancer: How are they linked?. Free Radic. Biol. Med..

[62] Wu J., Liu W., Koenig K., Idell S., Broaddus V.C. (2000). Vitronectin adsorption to chrysotile asbestos increases fiber phagocytosis and toxicity for mesothelial cells. Am. J. Physiol. Lung Cell. Mol. Physiol..

[63] Hillegass J.M., Miller J.M., MacPherson M.B., Westbom C.M., Sayan M., Thompson J.K., Macura S.L., Perkins T.N., Beuschel S.L., Alexeeva V. (2013). Asbestos and erionite prime and activate the NLRP3 inflammasome that stimulates autocrine cytokine release in human mesothelial cells. Part. Fibre Toxicol..

[64] Chow M.T., Tschopp J., Möller A., Smyth M.J. (2012). NLRP3 promotes inflammation-induced skin cancer but is dispensable for asbestos-induced mesothelioma. Immunol. Cell Biol..

[65] Petrilli V., Dostert C., Muruve D.A., Tschopp J. (2007). The inflammasome: A danger sensing complex triggering innate immunity. Curr. Opin. Immunol..

[66] Westbom C., Thompson J.K., Leggett A., MacPherson M., Beuschel S., Pass H., Vacek P., Shukla A. (2015). Inflammasome modulation by chemotherapeutics in malignant mesothelioma. PLoS ONE.

[67] Wang Y., Faux S.P., Hallden G., Kirn D.H., Houghton C.E., Lemoine N.R., Patrick G. (2004). Interleukin-1beta and tumour necrosis factor-alpha promote the transformation of human immortalised mesothelial cells by erionite. Int. J. Oncol..

[68] Antony V.B. (2003). Immunological mechanisms in pleural disease. Eur. Respir. J..

[69] Driscoll K.E., Maurer J.K., Higgins J., Poynter J. (1995). Alveolar macrophage cytokine and growth factor production in a rat model of crocidolite-induced pulmonary inflammation and fibrosis. J. Toxicol. Environ. Health.

[70] Driscoll K.E., Carter J.M., Howard B.W., Hassenbein D., Janssen Y.M., Mossman B.T. (1998). Crocidolite activates NF-kappa B and MIP-2 gene expression in rat alveolar epithelial cells. Role of mitochondrial-derived oxidants. Environ. Health Perspect..

[71] Bielefeldt-Ohmann H., Fitzpatrick D.R., Marzo A.L., Jarnicki A.G., Himbeck R.P., Davis M.R., Manning L.S., Robinson B.W. (1994). Patho- and immunobiology of malignant mesothelioma: Characterisation of tumour infiltrating leucocytes and cytokine production in a murine model. Cancer Immunol. Immunother..

[72] Landskron G., de la Fuente M., Thuwajit P., Thuwajit C., Hermoso M.A. (2014). Chronic inflammation and cytokines in the tumor microenvironment. J. Immunol. Res..

[73] Caja F., Vannucci L. (2015). TGFβ: A player on multiple fronts in the tumor microenvironment. J. Immunotoxicol..

[74] Zitvogel L., Kepp O., Galluzzi L., Kroemer G. (2012). Inflammasomes in carcinogenesis and anticancer immune responses. Nat. Immunol..

[75] Naldini A., Carraro F. (2005). Role of inflammatory mediators in angiogenesis. Curr. Drug Targets Inflamm. Allergy.

[76] Fox S.A., Loh S.S., Mahendran S.K., Garlepp M.J. (2012). Regulated chemokine gene expression in mouse mesothelioma and mesothelial cells: TNF-α upregulates both CC and CXC chemokine genes. Oncol. Rep..

[77] Rollins B.J. (2006). Inflammatory chemokines in cancer growth and progression. Eur. J. Cancer.

[78] Vlaeminck-Guillem V., Bienvenu J., Isaac S., Grangier B., Golfier F., Passot G., Bakrin N., RodriguezLafrasse C., Gilly F.N., Glehen O. (2013). Intraperitoneal cytokine level in patients with peritoneal surface malignancies. A study of the RENAPE (French Network for Rare Peritoneal Malignancies). Ann. Surg. Oncol..

[79] Comar M., Zanotta N., Bonotti A., Tognon M., Negro C., Cristaudo A., Bovenzi M. (2014). Increased levels of C-C chemokine RANTES in asbestos exposed workers and in malignant mesothelioma patients from an hyperendemic area. PLoS ONE.

[80] Xu J., Alexander D.B., Iigo M., Hamano H., Takahashi S., Yokoyama T., Kato M., Usami I., Tokuyama T., Tsutsumi M. (2015). Chemokine (C-C motif) ligand 3 detection in the serum of persons exposed to asbestos: A patient-based study. Cancer Sci..

[81] Terlizzi M., Casolaro V., Pinto A., Sorrentino R. (2014). Inflammasome: Cancer’s friend or foe?. Pharmacol. Therap..

[82] Dinarello C.A. (2010). Why not treat human cancer with interleukin-1 blockade?. Cancer Metastasis Rev..

[83] Izzi V., Chiurchiù V., Doldo E., Palumbo C., Tesoldi I., Bei R., Albonici L., Modesti A. (2013). Interleukin-17 produced by malignant mesothelioma-polarized immune cells promotes tumor growth and invasiveness. Eur. J. Inflamm..

[84] Miselis N.R., Lau B.W., Wu Z., Kane A.B. (2010). Kinetics of host cell recruitment during dissemination of diffuse malignant peritoneal mesothelioma. Cancer Microenviron..

[85] Veltman J.D., Lambers M.E., van Nimwegen M., Hendriks R.W., Hoogsteden H.C., Hegmans J.P., Aerts J.G. (2010). Zoledronic acid impairs myeloid differentiation to tumour-associated macrophages in mesothelioma. Br. J. Cancer.

[86] Veltman J.D., Lambers M.E., van Nimwegen M., Hendriks R.W., Hoogsteden H.C., Aerts J.G., Hegmans J.P. (2010). COX-2 inhibition improves immunotherapy and is associated with decreased numbers of myeloid-derived suppressor cells in mesothelioma. Celecoxib influences MDSC function. BMC Cancer.

[87] Yang H., Pellegrini L., Napolitano A., Giorgi C., Jube S., Preti A., Jennings C.J., de Marchis F., Flores E.G., Larson D. (2015). Aspirin delays mesothelioma growth by inhibiting HMGB1-mediated tumor progression. Cell Death Dis..

[88] Sica A., Allavena P., Mantovani A. (2008). Cancer related inflammation: The macrophage connection. Cancer Lett..

[89] Burt B.M., Rodig S.J., Tilleman T.R., Elbardissi A.W., Bueno R., Sugarbaker D.J. (2011). Circulating and tumor-infiltrating myeloid cells predict survival in human pleural mesothelioma. Cancer.

[90] Hegmans J.P., Hemmes A., Hammad H., Boon L., Hoogsteden H.C., Lambrecht B.N. (2006). Mesothelioma environment comprises cytokines and T-regulatory cells that suppress immune responses. Eur. Respir. J..

[91] Miselis N.R., Wu Z.J., van Rooijen N., Kane A.B. (2008). Targeting tumor-associated macrophages in an orthotopic murine model of diffuse malignant mesothelioma. Mol. Cancer Ther..

[92] Napolitano A., Pellegrini L., Dey A., Larson D., Tanji M., Flores E.G., Kendrick B., Lapid D., Powers A., Kanodia S. (2016). Minimal asbestos exposure in germline BAP1 heterozygous mice is associated with deregulated inflammatory response and increased risk of mesothelioma. Oncogene.

[93] Nishimura Y., Kumagai-Takei N., Matsuzaki H., Lee S., Maeda M., Kishimoto T., Fukuoka K., Nakano T., Otsuki T. (2015). Functional alteration of natural killer cells and cytotoxic T limphocytes upon asbestos exposure and in malignant mesothelioma patients. BioMed. Res. Int..

[94] Maeda M., Nishimura Y., Hayashi H., Kumagai N., Chen Y., Murakami S., Miura Y., Hiratsuka J., Kishimoto T., Otsuki T. (2011). Reduction of CXC chemokine receptor 3 in an *in vitro* model of continuous exposure to asbestos in a human T-cell line, MT-2. Am. J. Respira. Cell Mol. Biol..

[95] Maeda M., Nishimura Y., Hayashi H., Kumagai N., Chen Y., Murakami S., Miura Y., Hiratsuka J., Kishimoto T., Otsuki T. (2011). Decreased CXCR3 expression in CD4+ T cells exposed to asbestos or derived from asbestos-exposed patients. Am. J. Respira. Cell Mol. Biol..

[96] Maeda M., Chen Y., Hayashi H., Kumagai-Takei N., Matsuzaki H., Lee S., Nishimura Y., Otsuki T. (2014). Chronic exposure to asbestos enhances TGF-β1 production in the human adult T cell leukemia virus-immortalized T cell line MT-2. Int. J. Oncol..

[97] Miura Y., Nishimura Y., Katsuyama H., Maeda M., Hayashi H., Dong M., Hyodoh F., Tomita M., Matsuo Y., Uesaka A. (2006). Involvement of IL-10 and Bcl-2 in resistance against an asbestos-induced apoptosis of T cells. Apoptosis.

[98] Feskanich D., Ziegler R.G., Michaud D.S., Giovannucci E.L., Speizer F.E., Willett W.C., Colditz G.A. (2000). Prospective study of fruit and vegetable consumption and risk of lung cancer among men and women. J. Natl. Cancer Inst..

[99] Bazzano L.A., He J., Ogden L.G., Loria C.M., Vupputuri S., Myers L., Whelton P.K. (2002). Fruit and vegetable intake and risk of cardiovascular disease in US adults: The first National Health and Nutrition Examination Survey Epidemiologic follow-up study. Am. J. Clin. Nutr..

[100] Mennen L.I., Sapinho D., de Bree A., Arnault N., Bertrais S., Galan P., Hercberg S. (2004). Consumption of foods rich in flavonoids is related to a decreased cardiovascular risk in apparently healthy French women. J. Nutr..

[101] Chang H.W., Baek S.H., Chung K.W., Son K.H., Kim H.P., Kang S.S. (1994). Inactivation of phospholipase A2 by naturally occurring biflavonoid, ochnaflavone. Biochem. Biophys. Res. Commun..

[102] Kang H.K., Ecklund D., Liu M., Datta S.K. (2009). Apigenin, a non-mutagenic dietary flavonoid, suppresses lupus by inhibiting autoantigen presentation for expansion of autoreactive Th1 and Th17 cells. Arthritis Res. Ther..

[103] Chi Y.S., Kim H.P. (2005). Suppression of cyclooxigenase-2 expression of skin fibroblasts by wogonin, a plant flavones from Scutellaria radix. Prostaglandins Leukot. Essent. Fat. Acids.

[104] Liang Y.C., Huang Y.T., Tsai S.H., Lin-Shiau S.Y., Chen C.F., Lin J.K. (1999). Suppression of inducible cyclooxygenase and inducible nitric oxide synthase by apigenin and related flavonoids in mouse macrophages. Carcinogenesis.

[105] Hamalainen M., Nieminen R., Vuorela P., Heinonen M., Moilanen E. (2007). Anti-inflammatory effects of flavonoids: Genistein, kaempferol, quercetin, and daidzein inhibit STAT-1 and NF-kappaB activations, whereas flavone, isorhamnetin, naringenin, and pelargonidin inhibit only NF-kappaB activation along with their inhibitory effect on iNOS expression and NO production in activated macrophages. Mediat. Inflamm..

[106] Halliwell B. (2008). Are polyphenols antioxidants or pro-oxidants? What do we learn from cell culture and *in vivo* studies?. Arch. Biochem. Biophys..

[107] León-Gonzáles A.J., Auger C., Schini-Kerth V.B. (2015). Pro-oxidant activity of polyphenols and its implication on cancer chemoprevention and chemotherapy. Biochem. Pharmacol..

[108] Miller J.M., Thompson J.K., MacPherson M.B., Beuschel S.L., Westbom C.M., Sayan M., Shukla A. (2014). Curcumin: A double hit on malignant mesothelioma. Cancer Prev. Res..

[109] Satoh M., Takemura Y., Hamada H., Sekido Y., Kubota S. (2013). EGCG indices human mesothelioma cell death by inducing reactive oxygen species and autophagy. Cancer Cell Int..

[110] Ranzato E., Martinotti S., Magnelli V., Murer B., Biffo S., Mutti L., Burlando B. (2012). Epigallocathechin-3-gallate induces mesothelioma cell death via H2 O2-dependent T-type Ca2+ channel opening. J. Cell. Mol. Med..

[111] Valenti D., de Bari L., Manente G.A., Rossi L., Mutti L., Moro L., Vacca R.A. (2013). Negative modulation of mitochondrial oxidative phosphorylation by epigallocatechin-3 gallate leads to growth arrest and apoptosis in human malignant pleural mesothelioma cells. Biochim. Biophys. Acta.

[112] Lee Y.J., Im J.H., Lee D.M., Park J.S., Won S.Y., Cho M.K., Nam H.S., Lee Y.J., Lee S.H. (2012). Synergistic inhibition of mesothelioma cell growth by the combination of clofarabine and resveratrol involves Nrf2 downregulation. BMB Rep..

[113] Lee Y.J., Park I.S., Lee Y.J., Shim J.H., Cho M.K., Nam H.S., Park J.W., Oh M.H., Lee S.H. (2014). Resveratrol contributes to chemosensivity of malignant mesothelioma cells with activation of p53. Food Chem. Toxicol..

[114] Faraonio R., Vergara P., Di Marzo D., Pierantoni M.G., Napolitano M., Russo T., Cimino F. (2006). p53 suppresses the Nrf2-dependent transcription of antioxidant response genes. J. Biol. Chem..

[115] Pietrofesa R.A., Velalopoulou A., Albelda S.M., Christofidou-Solomidou M. (2016). Asbestos Induces Oxidative Stress and Activation of Nrf2 Signaling in Murine Macrophages: Chemopreventive Role of the Synthetic Lignan Secoisolariciresinol Diglucoside (LGM2605). Int. J. Mol. Sci..

[116] Kostyuk V.A., Potapovich A.I., Speransky S.D., Maslova G.T. (1996). Protective effect of natural flavonoids on rat peritoneal macrophages injury caused by asbestos fibers. Free Radic. Biol. Med..

[117] Kostyuk V.A., Potapovich A.I., Vladykovskaya E.N., Hiramatsu M. (2000). Protective effects of green tea catechins against asbestos-induced cell injury. Planta Med..

[118] Potapovich A.I., Kostyuk V.A. (2003). Comparative study of antioxidant properties and cytoprotective activity of flavonoids. Biochemistry.

[119] Grivennikov S.I., Greten F.R., Karin M. (2010). Immunity, inflammation, and cancer. Cell.

[120] Valko M., Leibfritz D., Moncol J., Cronin M.T., Mazur M., Telser J. (2007). Free radicals and antioxidants in normal physiological functions and human disease. Int. J. Biochem. Cell Biol..

[121] Cohen A.N., Veena M.S., Srivatsan E.S., Wang M.B. (2009). Suppression of interleukin 6 and 8 production in head and neck cancer cells with curcumin via inhibition of Ikappa beta kinase. Arch. Otolaryngol. Head Neck Surg..

[122] Wang Y., Yu C., Pan Y., Yang X., Huang Y., Feng Z., Li X., Yang S., Liang G. (2011). A novel synthetic mono-carbonyl analogue of curcumin, A13, exhibits anti-inflammatory effect *in vivo* by inhibition of inflammatory mediators. Inflammation.

[123] Serafini M., Peluso I., Raguzzini A. (2010). Flavonoids as anti-inflammatory agents. Proc. Nutr. Soc..

[124] Hirano T., Higa S., Arimitsu J., Naka T., Shima Y., Ohshima S., Fujimoto M., Yamadori T., Kawase I., Tanaka T. (2004). Flavonoids such as luteolin, fisetin and apigenin are inhibitors of interleukin-4 and interleukin-13 production by activated human basophils. Int. Arch. Allergy Immunol..

[125] Chen P.C., Wheeler D.S., Malhotra V., Odoms K., Denenberg A.G., Wong H.R. (2002). A green tea-derived polyphenol, epigallocatechin-3-gallate, inhibits IKappaB kinase activation and IL-8 gene expression in respiratory epithelium. Inflammation.

[126] Shin H.Y., Kim S.H., Jeong H.J., Kim S.Y., Shin T.Y., Um J.Y., Hong S.H., Kim H.M. (2007). Epigallocatechin-3- gallate inhibits secretion of TNF-alpha, IL-6 and IL-8 through the attenuation of ERK and NF-kappaB in HMC- 1 cells. Int. Arch. Allergy Immunol..

[127] Ahmed S., Marotte H., Kwan K., Ruth J.H., Campbell P.L., Rabquer B.J., Pakozdi A., Koch A.E. (2008). Epigallocatechin-3-gallate inhibits IL-6 synthesis and suppresses transsignaling by enhancing soluble gp130 production. Proc. Natl. Acad. Sci. USA.

[128] Rasheed Z., Anbazhagan A.N., Akhtar N., Ramamurthy S., Voss F.R., Haqqi T.M. (2009). Green tea polyphenol epigallocatechin-3-gallate inhibits advanced glycation end product-induced expression of tumor necrosis factor-alpha and matrix metalloproteinase-13 in human chondrocytes. Arthritis Res. Ther..

[129] Park H.H., Lee S., Son H.Y., Park S.B., Kim M.S., Choi E.J., Singh T.S., Ha J.H., Lee M.G., Kim J.E. (2008). Flavonoids inhibit histamine release and expression of proinflammatory cytokines in mast cells. Arch. Pharm. Res..

[130] Sun C., Hu Y., Liu X., Wu T., Wang Y., He W., Wei W. (2006). Resveratrol downregulates the constitutional activation of nuclear factor-kappaB in multiple myeloma cells, leading to suppression of proliferation and invasion, arrest of cell cycle, and induction of apoptosis. Cancer Genet. Cytogenet..

[131] Manna S.K., Mukhopadhyay A., Aggarwal B.B. (2000). Resveratrol suppresses TNF-induced activation of nuclear transcription factors NF-kappaB, activator protein-1, and apoptosis: Potential role of reactive oxygen intermediates and lipid peroxidation. J. Immunol..

[132] Kim G.Y., Kim K.H., Lee S.H., Yoon M.S., Lee H.J., Moon D.O., Lee C.M., Ahn S.C., Park Y.C., Park Y.M. (2005). Curcumin inhibits immunostimulatory function of dendritic cells: MAPKs and translocation of NF-kappaB as potential targets. J. Immunol..

[133] Garcia-Mediavilla M.V., Crespo I., Collado P.S., Esteller A., Sanchez-Campos S., Tunon M.J. (2007). Anti-inflammatory effect of the flavones quercetin and kaempferol in Chang Liver cells involves inhibition of inducible nitric oxide synthase, cyclooxygenase-2 and reactive C-protein, and down-regulation of the nuclear factor kappaB pathway. Eur. J. Pharmacol..

[134] Nicholas C., Batra S., Vargo M.A., Voss O.H., Gavrilin M.A., Wewers M.D., Guttridge D.C., Grotewold E., Doseff A.I. (2007). Apigenin blocks lipopolysaccharide-induced lethality *in vivo* and proinflammatory cytokines expression by inactivating NF-kappa B through the suppression of p65 phosphorylation. J. Immunol..

[135] Romier B., van de Walle J., During A., Larondelle Y., Schneider Y.J. (2008). Modulation of signaling nuclear factor-kappaB activation pathway by polyphenols in human intestinal Caco-2 cells. Br. J. Nutr..

[136] Lin R.W., Chen C.H., Wang Y.H., Ho M.L., Hung S.H., Chen I.S., Wang G.J. (2009). (−)-Epigallocatechin gallate inhibition of osteoclastic differentiation via NF-kappaB *Biochem*. Biophys. Res. Commun..

[137] Laua F.C., Josepha J.A., McDonald J.E., Kalt A.W. (2009). Attenuation of iNOS and COX2 by blueberry polyphenols is mediated through the suppression of NF-κB activation. J. Funct. Foods.

[138] Kim J.W., Jin Y.C., Kim Y.M., Rhie S., Kim H.J., Seo H.G., Lee J.H., Ha Y.L., Chang K.C. (2009). Daidzein administration *in vivo* reduces myocardial injury in a rat ischemia/reperfusion model by inhibiting NF-κB activation. Life Sci..

[139] Bharti A.C., Donato N., Aggarwal B.B. (2003). Curcumin (diferuloylmethane) inhibits constitutive and IL-6-inducible STAT3 phosphorylation in human multiple myeloma cells. J. Immunol..

[140] Chakravarti N., Myers J.N., Aggarwal B.B. (2006). Targeting constitutive and interleukin-6-inducible signal transducers and activators of transcription 3 pathway in head and neck squamous cell carcinoma cells by curcumin (diferuloylmethane). Int. J. Cancer.

[141] Wung B.S., Hsu M.C., Wu C.C., Hsieh C.W. (2005). Resveratrol suppresses IL-6- induced ICAM-1 gene expression in endothelial cells: Effects on the inhibition of STAT3 phosphorylation. Life Sci..

[142] Masuda M., Suzui M., Weinstein I.B. (2001). Effects of epigallocatechin-3- gallate on growth, epidermal growth factor receptor signaling pathways, gene expression, and chemosensitivity in human head and neck squamous cell carcinoma cell lines. Clin. Cancer Res..

[143] Kadariya Y., Menges C.W., Talarchek J., Cai K.Q., Klein-Szanto A.J., Pietrofesa R.A., Christofidou-Solomidou M., Cheung M., Mossman B.T., Shukla A., Testa J.R. (2016). Inflammation-Related IL-1β/IL-1R Signaling Promotes the Development of Asbestos-Induced Malignant Mesothelioma. Cancer Prev. Res..

[144] Miao E.A., Rajan J.V., Aderem A. (2011). Caspase-1-induced pyroptotic cell death. Immunol. Rev..

[145] Wang Y., Rishi A.K., Wu W., Polin L., Sharma S., Levi E., Albelda S., Pass H.I., Wali A. (2011). Curcumin suppresses growth of mesothelioma cells *in vitro* and *in vivo*, in part, by stimulating apoptosis. Mol. Cell. Biochem..

[146] Cioce M., Canino C., Pulito C., Muti P., Strano S., Blandino G. (2012). Butein impairs the protumorigenic activity of malignant pleural mesothelioma cells. Cell Cycle.

[147] Dabir S., Kluge A., Dowlati A. (2009). The association and nuclear translocation of the PIAS3-STAT3 complex is ligand and time dependent. Mol. Cancer Res..

[148] Dabir S., Kluge A., Kresak A., Yang M., Fu P., Groner B., Wildey G., Dowlati A. (2014). Low PIAS3 expression in malignant mesothelioma is associated with increased STAT3 activation and poor patient survival. Clin. Cancer Res..

[149] Martinotti S., Ranzato E., Burlando B. (2011). *In vitro* screening of synergistic ascorbate-drug combinations for the treatment of malignant mesothelioma. Toxicol. Vitro.

[150] Martinotti S., Ranzato E., Parodi M., Vitale M., Burlando B. (2014). Combination of ascorbate/epigallocatechin-3-gallate/gemcitabine synergistically induces cell cycle deregulation and apoptosis in mesothelioma cells. Toxicol. Appl. Pharmacol..

[151] Beishline K., Azizkhan-Clifford J. (2015). Sp1 and the ‘hallmarks of cancer’. FEBS J..

[152] Lee K.A., Lee Y.J., Ban J.O., Lee Y.J., Lee S.H., Cho M.K., Nam H.S., Hong J.T., Shim J.H. (2012). The flavonoid resveratrol suppresses growth of human malignant pleural mesothelioma cells through direct inhibition of specificity protein 1. Int. J. Mol. Med..

[153] Lee Y.J., Lee Y.J., Im J.H., Won S.Y., Kim Y.B., Cho M.K., Nam H.S., Choi Y.J., Lee S.H. (2013). Synergistic anti-cancer effects of resveratrol and chemotherapeutic agent clofarabine against human malignant mesothelioma MSTO-211H cells. Food Chem. Toxicol..

[154] Chae J.I., Cho J.H., Lee K.A., Choi N.J., Seo K.S., Kim S.B., Lee S.H., Shim J.H. (2012). Role of transcriptor factor Sp1 in the quercetin-mediated inhibitory effect on human malignant pleural mesothelioma. Int. J. Mol. Med..

[155] Chae J.I., Jeon Y.J., Shim J.H. (2013). Downregulation of Sp1 is involved in honokiol-induced cell cycle arrest and apoptosis in human malignant pleural mesothelioma cells. Oncol. Rep..

[156] Kim K.H., Yoon G., Cho J.J., Cho J.H., Cho Y.S., Chae J.I., Shim J.H. (2015). Licochalcone A induces apoptosis in malignant pleural mesothelioma through downregulation of Sp1 and subsequent activation of mitochondria-related apoptotic pathway. Int. J. Oncol..

[157] Lee K.A., Lee S.H., Lee Y.J., Baeg S.M., Shim J.H. (2012). Hesperidin induces apoptosis by inhibiting Sp1 and its regulatory protein in MSTO-211H cells. Biomol. Ther..

[158] Lee K.A., Chae J.I., Shim J.H. (2012). Natural diterpenes from coffee, cafestol and kahweol induce apoptosis through regulation of specificity protein 1 expression in human malignant pleural mesothelioma. J. Biomed. Sci..

[159] Roomi M.W., Ivanov V., Kalinovsky T., Niedzwiecki A., Rath M. (2006). Inhibition of malignant mesothelioma cell matrix metalloproteinase production and invasion by a novel nutrient mixture. Exp. Lung Res..

